# Temporally Correlated Stochastic Spiking Neural Networks Enabled by 2D Ferroelectric Synapse‐Neuron Integration

**DOI:** 10.1002/advs.78300

**Published:** 2026-10-14

**Authors:** Hanseo Jeon, Ui Do Ji, Sangmin Lee, Gihyeon Bhin, Chang Yong Park, Ji‐Sang Park, Byungchul Jang, Sungjoo Lee

**Affiliations:** ^1^ SKKU Advanced Institute of Nanotechnology (SAINT) Sungkyunkwan University Suwon South Korea; ^2^ Department of Nano Science and Technology Sungkyunkwan University Suwon South Korea; ^3^ School of Electronic and Electrical Engineering Kyungpook National University Daegu South Korea

**Keywords:** 2D ferroelectric, stochastic neurons, stochastic spike neural network, synapses, temporally correlated

## Abstract

Stochastic neural computation is essential for probabilistic inference and brain‐inspired information processing. However, most hardware stochastic neurons generate statistically independent spikes and fail to reproduce the temporal correlations observed in biological neurons. We demonstrate an integrated hardware synapse‐neuron consisting of a FeFET synapse and a CulnP_2_S_6_ neuron, where the neuron exhibits temporally correlated stochastic spiking. The spike probability is continuously tuned by synaptic weight through voltage modulation, enabling hardware stochastic neural computation. Electrical measurements reveal voltage‐dependent stochastic firing accompanied by leaky‐integration‐firing dynamics. Structural and polarization analyses reveal differences in Cu‐related atomic contrast and partially retained polarization states, suggesting a history‐dependent internal state. Autocorrelation and inter‐spike interval analyses reveal temporal dependencies between successive stochastic spikes, which are consistent with the evolution of this retained state, with the strongest correlation emerging in the intermediate stochastic regime. The proposed stochastic spiking neural network achieves an inference accuracy of approximately 90% and maintains over 87% accuracy under severe noise conditions, demonstrating the computational advantages of temporally correlated stochastic spiking for robust probabilistic neural computation. This work demonstrates a class of 2D ferroelectric neuromorphic hardware that inherently incorporates intrinsic temporal dynamics, providing a pathway toward next‐generation neuromorphic and probabilistic computing.

## Graphical abstract

We demonstrate a stochastic spiking neural network by integrating an α‐ln_2_Se_3_/h‐BN/CIPS FeFET synapse with an Au/CIPS/Au neuron. Unlike conventional memoryless stochastic neurons, the proposed neuron generates stochastic spikes through the evolution of internal ferroelectric polarization states, giving rise to intrinsic temporal correlations and history‐dependent dynamics. Polarization‐state evolution and incomplete polarization relaxation are associated with the formation of a history‐dependent internal state, which continuously influences subsequent stochastic switching events and provides a plausible physical mechanism for temporally correlated stochastic spiking.

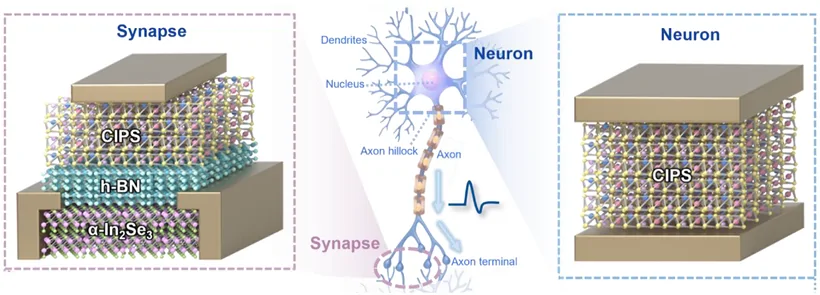

## 1 Introduction

Biological neurons exhibit inherently stochastic firing behavior rather than deterministic responses. Even under identical input conditions, neuronal spiking occurs probabilistically due to factors such as thermal fluctuations of ion channels, synaptic variability, and internal states [1, 2, 3, 4]. This stochastic firing plays a crucial role in sensory information processing, learning, and decision‐making [4, 5]. In particular, it enables efficient exploration of multiple possibilities under intrinsic uncertainty, thereby facilitating flexible and robust inference [4, 5]. Motivated by these biological characteristics, recent advances in neuromorphic computing have shifted beyond conventional deterministic neuron models toward the development of stochastic neurons. By enabling probabilistic spike generation, stochastic neurons support probabilistic inference and provide robust information processing in noisy environments [4, 6, 7, 8]. Furthermore, stochastic neural dynamics have recently attracted significant attention as a hardware‐efficient means of implementing probabilistic computing paradigms, including Bayesian inference, uncertainty‐aware learning, and generative AI models [9, 10]. Therefore, physically realizing the intrinsic stochasticity of biological neurons has emerged as a key challenge for achieving efficient information processing and enhanced inference performance in neuromorphic systems.

However, most previously proposed stochastic neurons primarily focus on the probability of spike occurrence, without considering the temporal correlations between individual spike events [4, 11, 12, 13]. In other words, although these neurons can generate spikes probabilistically, previous firing events do not influence subsequent firing events, and thus temporal correlations are absent between successive firing events, a characteristic defined as “memoryless” stochastic firing. Such stochastic neurons have been implemented using a variety of physical devices, including memristors [4, 14], phase‐change materials [13], magnetic tunnel junctions [11, 15, 16], and Schottky diodes [12]. These devices exploit intrinsic stochastic switching characteristics to realize probabilistic neuron behavior, typically exhibiting a sigmoid‐like relationship between the applied voltage and spike probability. While these approaches successfully enable stochastic spiking, the generated spike events are typically temporally uncorrelated because the stochastic switching is governed primarily by instantaneous input conditions rather than the accumulated internal state of the neuron. In contrast, biological neurons retain a portion of their internal state even after spike generation, rather than being fully reset. This residual internal state influences both the probability and timing of subsequent spikes, leading to the formation of temporal correlations between spike events [17, 18]. Such temporally correlated stochasticity is considered essential for biological neural systems, as it enables adaptive temporal integration, history‐dependent information encoding, and efficient processing of sequential data. As a result, existing stochastic neurons, despite enabling probabilistic spike generation, remain limited in capturing sequential spike dependencies and retained internal‐state dynamics. Furthermore, most previously reported stochastic neurons focus on probabilistic spike generation at the individual device, while hardware implementations integrating synaptic and neuronal functionalities remain limited. Such functional separation introduces additional circuit complexity and energy consumption caused by data movement, hindering the efficient emulation of integrated information processing in biological neural systems.

In this work, to overcome these limitations, we propose a hardware approach for implementing probabilistic neural networks by leveraging the internal‐state dynamics of ferroelectric materials. During repeated electrical stimulation, the polarization state established in CulnP_2_S_6_ (CIPS) is not completely reset after each spike but partially persists into the subsequent excitation cycle [19]. This residual internal state is superimposed with newly developed polarization during the next stimulation, thereby introducing sequential dependencies between successive spike events. Consequently, the probability and timing of spike generation become influenced by preceding spike activity [17, 20], enabling probabilistic spike generation governed by accumulated polarization states. Unlike conventional stochastic devices that rely solely on instantaneous random switching events, the proposed approach utilizes accumulated ferroelectric internal states as a physical origin of temporally correlated stochasticity. We demonstrate a stochastic neuron exhibiting temporally correlated spike generation associated with accumulated polarization states. Based on this accumulated‐state‐driven stochasticity, we integrate a FeFET‐based synapse with a CIPS‐based stochastic neuron based on the van der Waals (vdW) ferroelectric material CIPS [8, 21], thereby realizing a hardware synapse‐neuron system capable of generating temporally correlated stochastic spikes. In this architecture, the FeFET synapse [22] stores analog synaptic weights, while the CIPS neuron converts the synaptic input into temporally correlated stochastic spikes through retained polarization dynamics. This hardware integration enables both synaptic plasticity and sequential stochastic neural processing within a unified neuromorphic framework. In this architecture, the temporal evolution of retained polarization states governs stochastic spike generation while enabling sequential neural information processing in SSNN. Furthermore, this hardware integration enables both synaptic plasticity and sequential stochastic neural processing within a neuromorphic system. In this framework, the balance between probabilistic spike generation and sequential spike dependency can be dynamically tuned via the applied voltage to optimize inference performance. Importantly, the strongest temporal correlation does not emerge under fully stochastic or fully deterministic conditions, but rather at an intermediate stochastic regime where stochastic switching and retained internal polarization coexist. By integrating the synapse and neuron in hardware to generate temporally correlated stochastic spikes and incorporating these characteristics into a Bayesian SSNN, we establish a neuromorphic platform that simultaneously supports computation and temporal information processing.

## 2 Results and Discussion

### 2.1 Hardware Synapse‐Neuron Integration and Temporally Correlated Stochastic Spike

Figure 1a illustrates a schematic of spike‐based signal transmission in a biological neuron and synapse. A biological neuron consists of dendrites, somas, and axons, where input signals are delivered to the dendrites through synaptic terminals and are temporally integrated in the soma. When the membrane potential exceeds a threshold, a spike is generated [23]. Importantly, these spike events occur stochastically rather than deterministically, reflecting the inherently stochastic nature of biological neuronal activity [24]. To emulate biological neural systems, we implemented both synaptic and neuronal components in hardware, where stochastic spike generation in the neuron is realized based on the intrinsic ferroelectric properties of CIPS. This presents a schematic of the hardware implementation of the synapse and neuron. The synapse is based on an α‐In_2_Se_3_/h‐BN/CIPS FeFET structure, where synaptic weights are modulated depending on the input signals. The neuron is implemented using an Au/CIPS/Au structure. The weighted output from the synapse is converted into a voltage signal through a R_load_ and subsequently delivered to the neuron input. The neuron integrates the weight input signal and generates stochastic spike outputs (Figure S1). The detailed fabrication process is discussed in Figures S2 and S3. Figure 1b schematically illustrates temporally correlated stochastic spike generation and synaptic‐weight‐dependent spike probability modulation. Although spike generation remains stochastic under identical input pulse conditions, the internal state established by previous spike events influences subsequent firing behavior, giving rise to temporal correlations between successive spikes. In addition, synaptic weights are converted into input voltages, enabling continuous tuning of spike probability. Figure 1c illustrates the physical origin of temporally correlated stochastic spikes. During the initial pulse, partial polarization switching gradually accumulates the internal polarization state. However, the accumulated polarization remains insufficient to stablish a transient high‐conductance state, resulting in negligible current generation, corresponding to integration. A portion of the polarization remains after partial relaxation, preserving the internal state created by the previous stimulation. During subsequent stimulation, newly formed polarization develops on the retained polarization state, further evolving the internal state. Once the accumulated polarization becomes sufficient to establish a transient high‐conductance state, abrupt current‐generation firing occurs. After firing, partial relaxation of the accumulated polarization reduces the internal state, corresponding to leaky behavior, returning the device toward a low‐conductance state and enabling subsequent stochastic firing cycles.

**FIGURE 1:**
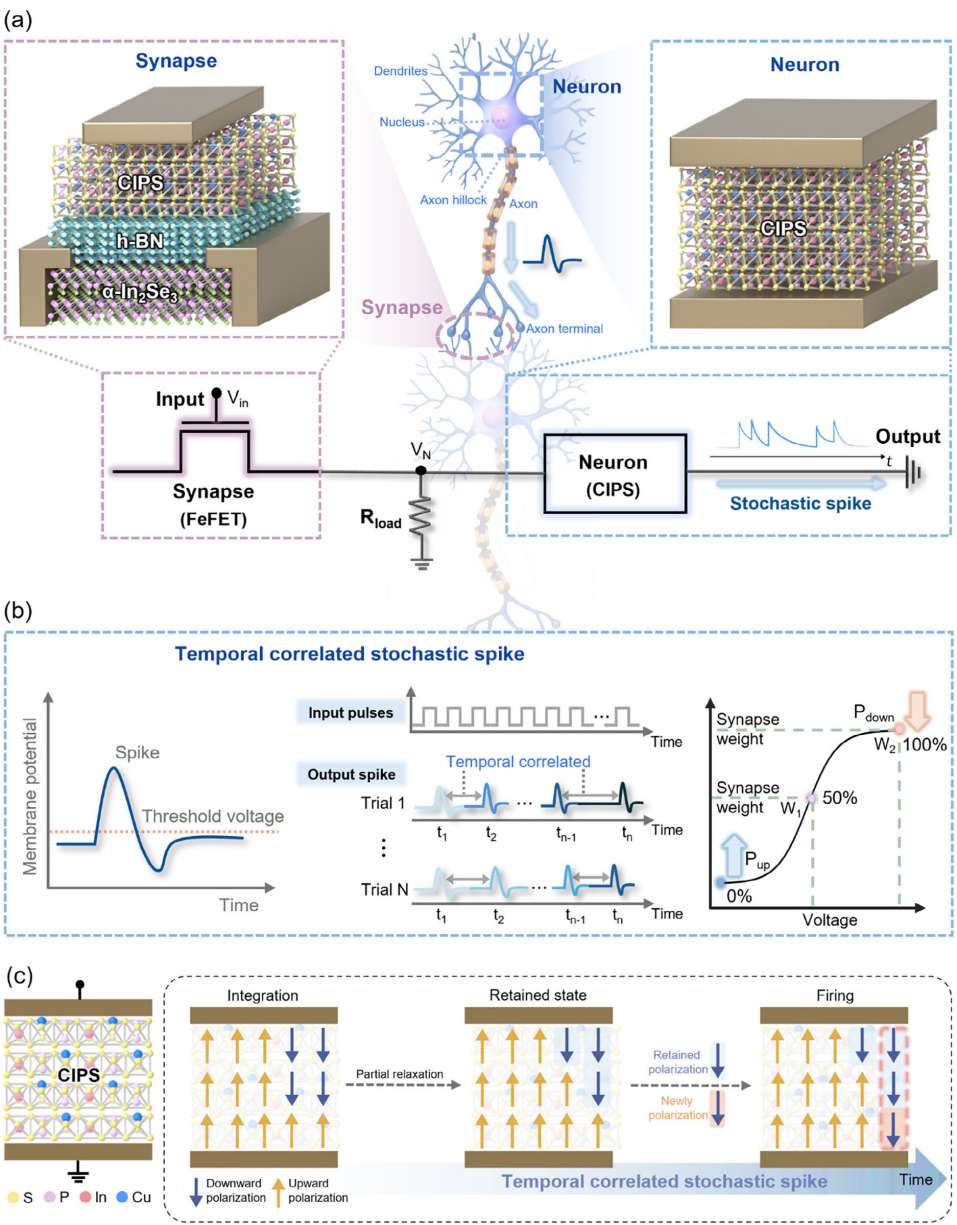
Hardware synapse‐neuron integrated enabling temporally correlated stochastic spike generation. (a) Schematic representations of the FeFET synapse and CIPS stochastic neuron, which form an integrated hardware synapse‐neuron for temporally correlated SSNN that emulates biological neural information processing. (b) Schematic illustration of temporally correlated stochastic spike generation and synaptic weight‐dependent firing probability. (c) Schematic illustration of the polarization‐state evolution and residual polarization associated with temporally correlated stochastic spike generation in CIPS.

### 2.2 Stochastic Spike Generation with Voltage‐Dependent Firing Probability

To experimentally investigate stochastic firing behavior in the proposed neuron, the electrical characteristics of the Au/CIPS/Au device were evaluated under repeated voltage‐pulse stimulation. Figure 2a illustrates the CIPS‐based stochastic spiking neuron. Prior to stochastic firing measurements, the electrical stability, and ferroelectric switching properties (Figures S4–S6) and uniform elemental distributions (Figure S7) of the device were confirmed. Figure 2b presents a representative current response measured under repeatedly applied 4.0 V pulses with a pulse width of 5 ms. The orange dashed line indicates the threshold current (I_th_) used for spike detection. A firing event was defined as an abrupt increase in current associated with the formation of a transient high‐conductance state. For quantitative analysis, a threshold current was set to 0.14 µA, corresponding to the mean value of the stochastic current distribution measured under the 4.0 V pulse. Notably, repeated piezo‐response force microscopy (PFM) measurements performed after applying 4.0 V pulses revealed a nearly balanced distribution of upward and downward polarization domains (Figure S8). This represents a stochastic operating regime in which firing and non‐firing events coexist. Therefore, the average current obtained under this condition was used as the criterion for spike detection. Figure 2c presents an enlarged view of the blue‐boxed region highlighted in Figure 2b. The magnified current response clearly reveals the leaky‐integration‐firing process preceding stochastic firing. At the initial state of pulse stimulation, the current remains low because the applied voltage is insufficient to immediately trigger stochastic firing. As repeated pulses evolve, the current gradually increases, indicating the progressive accumulation of an internal state (integration). In certain instances, the current decreases even while the voltage pulse is actively applied. This behavior is attributed to partial relaxation of the accumulated internal state during stimulation (leaky). As the accumulated polarization approaches the threshold required for transient high‐conductance state formation, small variations in polarization evolution influence whether a transient high‐conductance state is established during a given pulse. Once a transient high‐conductance state is established, an abrupt increase in current is observed, which is defined as a firing event. Consequently, firing occurs probabilistically even under identical input conditions. These observations indicate that stochastic firing is not solely determined by the instantaneous applied voltage but is influenced by an internal state that continuously evolves during repeated electrical stimulation. Figure 2d presents representative stochastic firing responses measured under different voltage conditions. As the voltage pulse increases from 3.8 to 4.0 and 4.2 V, the density of firing events gradually increases. Sparse stochastic spike generation is observed at 3.8 V, whereas a near‐continuous spiking regime emerges at 4.2 V. These results suggest that the stochastic firing probability can be modulated by the applied voltage. To quantitatively evaluate this voltage dependence, Figure 2e presents the spike probability as a function of the applied voltage. Voltage pulses were varied from 3.8 to 4.2 V in increments of 0.05 V. For each voltage condition, spike probability was determined from 50 applied pulses per cycle, and the measurement was repeated for 10 cycles. The error bars represent the standard deviation of the spike probability across the 10 cycles. As the voltage increases, the spike probability evolves from near 0 to near 1 with increasing voltage, exhibiting a characteristic sigmoidal dependence. The stochastic current fluctuations measured at each voltage condition are summarized in Figure S9.

**FIGURE 2:**
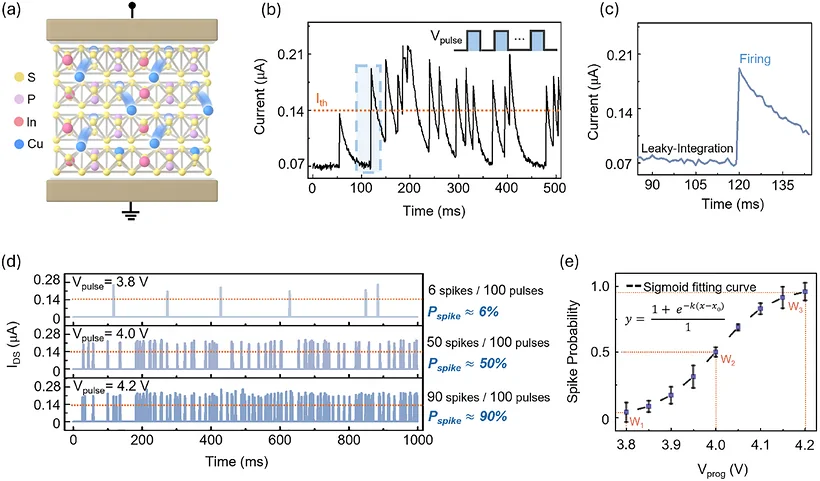
Stochastic spike generation with voltage‐dependent firing probability in the CIPS neuron. (a) Schematic illustration of the Au/CIPS/Au stochastic neuron structure. (b) Stochastic spike current response under repeated voltage pulse stimulation. (c) Enlarged view of a single spike event showing leaky‐integration and firing behavior. (d) Stochastic spike dynamics under different pulse voltage conditions. Spike probabilities of approximately 6%, 50%, and 90% are observed at V_pulse_ = 3.8, 4.0, and 4.2 V, respectively. (e) Voltage‐dependent spike probability and corresponding sigmoid fitting curve. Synaptic weight states (W_1_–W_3_) are mapped onto different stochastic spike probabilities.

Notably, an approximately 50% spike probability is observed at 4.0 V, where firing and non‐firing events coexist. These results demonstrate that the spike probability can be continuously tuned through the applied voltage. This voltage‐dependent probability modulation enables integration with hardware synapses. The conductance of the FeFET synapse is converted into input voltages through a load resistor and subsequently applied to the stochastic neuron. Consequently, different synaptic weight states (W_1_, W_2_, W_3_) are mapped to different input voltages, resulting in distinct firing probabilities. The synaptic characteristics of the FeFET synapse is presented in Figure S10 [22], while its retention and endurance characteristics of the FeFET synapse are presented in Figure S11.

To verify device‐to‐device reproducibility, the voltage‐dependent sigmoid characteristics were measured across 50 devices (Figure S12), showing consistent stochastic spiking behavior. Furthermore, temperature‐dependent spike probability measurements were performed (Figure S13). The sigmoid curve shifts with temperature, suggesting that stochastic spiking is closely related to thermally activated evolution of the internal state.

At 4.0 V, the energy associated with each stochastic firing event was estimated to be approximately 2.8 nJ/spike using Equation (1).

(1) $$E_{\mathrm{spike}}=V_{\mathrm{spike}}\times \;I_{\mathrm{th}}\times \;t_{\mathrm{spike}}$$

In addition, the first‐spike latency was evaluated as an indirect indicator of inference latency, as shown in Figure S14. The average first‐spike latency decreased from 87 ms at 3.8 V to 30 ms at 4.0 V and 7 ms at 4.2 V, demonstrating voltage‐tunable stochastic firing dynamics. These energy and latency values represent the present proof‐of‐concept device operated with ms‐scale excitation pulses rather than an optimized neuromorphic implementation. Further reduction in the active device dimensions and pulse duration is therefore expected to reduce both the energy consumption and firing latency.

### 2.3 History‐Dependent Internal States Underlying Temporally Correlation Stochastic Spike

The observed stochastic firing behavior suggests the existence of an internal state. To investigate the internal‐state in CIPS evolution associated with electrical stimulation, scanning transmission electron microscopy (STEM) and PFM measurements were performed. Figure 3a and b show STEM images from different CIPS samples representing the pristine and voltage‐applied states, respectively, together with the corresponding intensity line profiles. Atomic‐column assignments were performed based on the previous reported CIPS crystal structure viewed along the (100) direction and the corresponding relative intensity distribution, which is consistent with previous reports in which atomic species were identified using atomic arrangement and intensity profiles in STEM of CIPS [25]. The blue circles indicate the assigned Cu‐columns. Intensity peaks are observed at the assigned Cu‐column positions in the corresponding line profiles, supporting the atomic‐column assignment. Comparison of the pristine and voltage‐applied samples reveals differences in the Cu‐related atomic contrast. To directly investigate the evolution of the internal state under electrical stimulation, in situ PFM measurements were performed under different electrical conditions. Figure 3c shows a 3 µm × 6 µm PFM phase image measured in the pristine state without electrical stimulation (0 V). The image exhibits a relatively uniform polarization state. After applying 4 V pulses (Figure 3d), mixed upward and downward polarization domains emerge, indicating electric‐field‐induced polarization‐state evolution. After allowing the device to relax for 20 min (Figure 3e), partial recovery toward the pristine state is observed. However, residual polarization domains remain visible, indicating that the electrically induced polarization state is only partially relaxed. Upon reapplying 4 V pulses (Figure 3f), the mixed‐domain configuration reappears, demonstrating that the polarization state can be repeatedly established while retaining a portion of the previously formed polarization state. This observation indicates that a portion of the electrically induced polarization state persists even after removal of the external stimulus. The voltage‐dependent retention of switched polarization further confirms the persistence of residual polarization states after electrical stimulation (Figure S15). These results demonstrate that polarization dynamics in CIPS exhibit partial relaxation and residual polarization, supporting the presence of a history‐dependent internal state under repeated electrical stimulation. Such polarization‐state evolution and residual polarization provide a plausible physical basis for the temporally correlated stochastic spike generation observed in our neuron.

**FIGURE 3:**
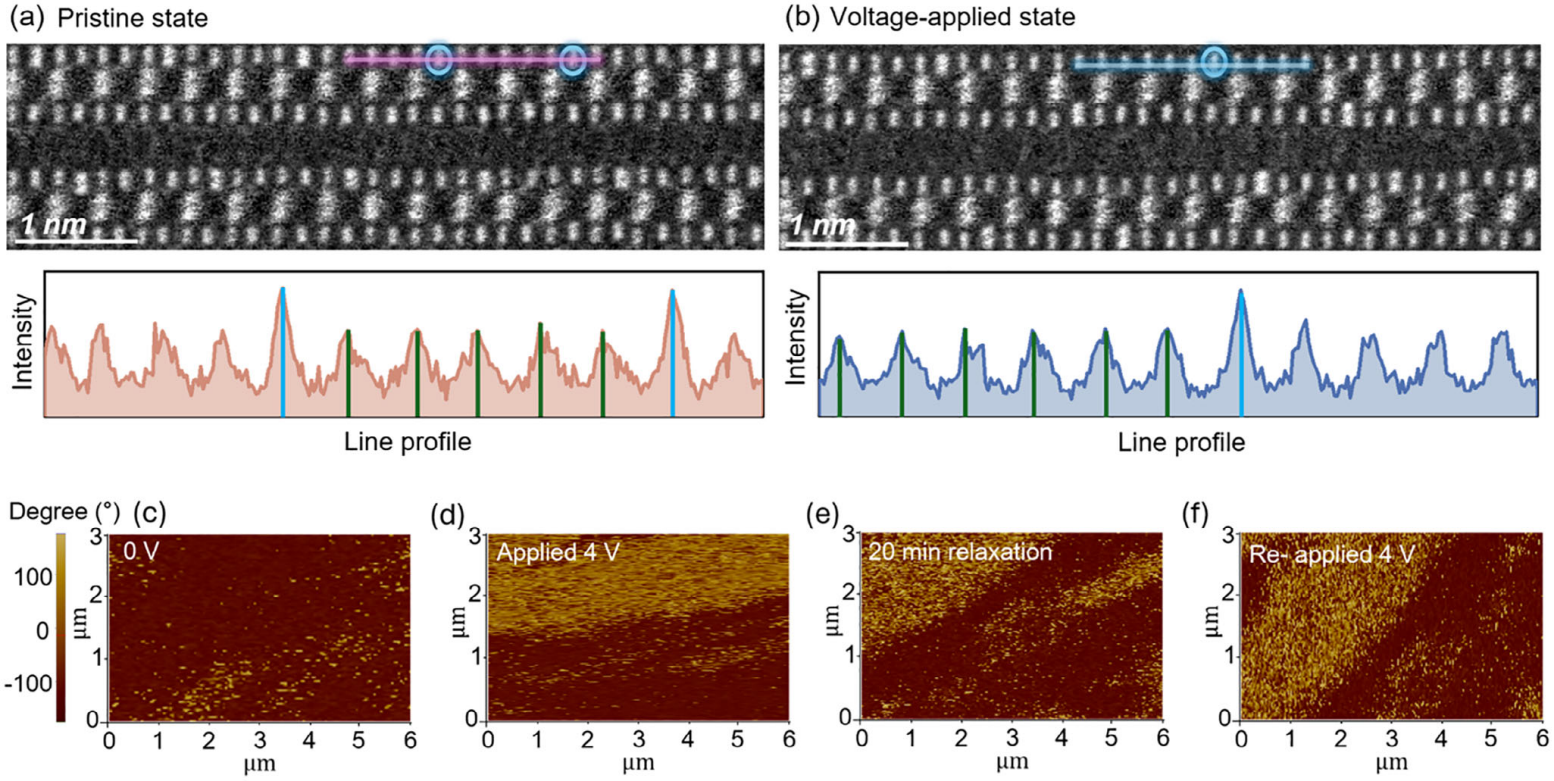
History‐dependent internal states in the CIPS neuron. (a, b) Atomic‐resolution STEM images of the pristine and voltage‐applied CIPS. Blue circles indicate the assigned Cu‐columns in the STEM images. The line profiles were extracted along the pink and blue lines in STEM, respectively. In the line profiles, the blue and green lines correspond to the assigned Cu‐columns and S‐columns, respectively. (c) PFM phase image of the pristine state (0 V). (d) PFM phase image after applying 4 V pulses. (e) PFM phase image after 20 min of relaxation following 4 V pulse application. (f) PFM phase image after reapplying 4 V pulses.

These results demonstrate that polarization dynamics in CIPS are partially irreversible and history dependent, enabling accumulated internal‐state evolution under repeated electrical stimulation. Such accumulated internal states provide the physical basis for temporally correlated stochastic spike generation in our neuron.

### 2.4 Temporally Correlation Analysis of Stochastic Spike

Having identified the existence of a history‐dependent internal polarization state in Figure 3, we next investigated whether this retained internal state produces temporal correlations in stochastic spike generation. To evaluate temporal correlations, autocorrelation function (ACF) and inter‐spike interval (ISI) analyses were performed. Both analyses were based on the current‐time responses obtained from the repeated pulse measurements shown in Figure 2d. Figure 4a schematically illustrates temporally correlated stochastic spiking, where the preceding inter‐spike interval (Δt_n_) influences the subsequent inter‐spike interval (∆t_n+1_) through retained internal states. For the ACF analysis, spike occurrence was determined by identifying whether the current exceeded the spike‐detection threshold (I_th_ = 0.14 µA) during each pulse. Pulses in which a spike occurred were assigned a value of 1, whereas pulses without a spike were assigned a value of 0, thereby generating a binary spike train. The ACF was calculated by evaluating the Pearson correlation coefficient (Γ) between a spike at a given time point and spikes occurring after a certain time delay. The time lag represents the time interval between the current pulse and pulses occurring *n* steps later. Therefore, the ACF was used to evaluate whether spike occurrence remains correlated over pulse intervals. Figure 4b presents the ACF calculated from stochastic spike trains measured at different voltages. At 3.8 V, the ACF rapidly decays toward zero with increasing lag, indicating that individual spike events occur nearly independently with minimal temporal correlation. In contrast, the ACF measured at 4.0 V decays more gradually than that at 3.8 V, indicating that spike occurrence remains correlated over pulse intervals. At 4.2 V, the ACF decreases more gradually with increasing time lag than the ACFs measured at 3.8 and 4.0 V, indicating stronger spike‐occurrence correlations over longer pulse intervals. To further examine temporal correlations in stochastic spike generation, the relationship between consecutive inter‐spike intervals (ISI_n_ and ISI_n+1_) was analyzed. For the ISI analysis, spike occurrence times extracted from current‐time response data were used. For each spike, the interval from the preceding spike and the interval to the subsequent spike were defined as ISI_n_ and ISI_n+1_, respectively. The correlation between ISI_n_ and ISI_n+1_ was then quantified using the Γ, which is given by:

(2) $$\Gamma =\frac{\sum_{i=1}^{N}\left(ISI_{n,i}-\bar{ISI_{n}}\right)\left(ISI_{n+1,i}-\bar{ISI_{n+1}}\right)}{\sqrt{\sum_{i=1}^{N}{\left(ISI_{n,i}-\bar{ISI_{n}}\right)}^{2}}\sum_{i=1}^{N}{\left(ISI_{n+1,i}-\bar{ISI_{n+1}}\right)}^{2}}$$

where Γ quantifies how strongly the timing of one spike interval is associated with the timing of the next spike interval. At 3.8 V, ISI_n_ is broadly distributed over a range of 0–100 ms, while ISI_n+1_, extends up to approximately 60 ms (Figure 4c). The ISI data points are widely scattered without a pronounced clustering feature, resulting in a relatively weak Pearson correlation coefficient of Γ = 0.285. This weak temporal correlation indicates that successive spike intervals are only weakly dependent on one another. In contrast, at 4.0 V, Figure 4d, ISI_n_ and ISI_n+1_ are distributed within the ranges of 0–60 and 0–55 ms, respectively, and exhibit a pronounced cluster concentrated within the highlighted blue region. Consequently, the Γ increases to Γ = 0.43, indicating that the interval of a given spike becomes more predictive of the interval of the following spike. Notably, this voltage corresponds to the regime identified in Figure 3d, where partially retained polarization states remain after electrical stimulation. The resulting history‐dependent internal state enhances the dependency between consecutive spike intervals. At 4.2 V, ISI_n_ and ISI_n+1_ are confined within narrower ranges of 0–35 ms and 0–15 ms, respectively (Figure 4e). A concentrated cluster is also observed within the highlighted region; however, the cluster is compressed into narrower ISI range compared with that at 4.0 V. As a result, the Pearson correlation coefficient decreases to Γ = 0.18. This reduction suggests that the interval variability becomes compressed at high firing probabilities, reducing the predictive relationship between consecutive spike intervals. These results further support that the strongest temporal correlation emerges in the intermediate voltage regime where switching and retained polarization coexist. The correlated ISI behavior was reproducibly observed across devices (Figure S16). This tendency is further reflected in the ISI distributions shown in Figure 4f–h, where the relative frequency represents the proportion of spikes belonging to each ISI range. While the ISI correlation analysis quantifies the temporal dependency between consecutive spike intervals, the ISI distribution provides information on the overall distribution of spike intervals under different voltage conditions. Together, these analyses characterize both the correlation and distribution properties of stochastic spike timing. At 3.8 V, ISIs are distributed over a broad range extending up to approximately 100 ms, indicating highly variable spike timing (Figure 4f). At 4.0 V, the ISIs distribution shifts toward shorter intervals while remaining distributed across an intermediate range extending to approximately 60 ms (Figure 4g). In contrast, at 4.2 V, ISIs are concentrated within a shorter interval range below approximately 40 ms, indicating reduced spike‐timing variability despite frequent firing events (Figure 4h). These results provide complementary information to the ISI correlation analysis by revealing how the distribution of spike intervals evolves with the applied voltage. The temporal correlation was also modulated by pulse width, further supporting the accumulation‐driven origin of correlated stochastic spiking (Figure S17). Notably, the strongest temporal correlation emerges in the intermediate voltage regime (4.0 V), where stochastic switching and retained internal states coexist. Although other mechanisms, such as charge trapping/detrapping and thermal effects, may also contribute to the observed temporal correlation, these mechanisms alone do not readily explain the non‐monotonic voltage dependence, with the strongest temporal correlation occurring at 4.0 V rather than at the highest applied voltage. These observations, together with the results presented in Figure 2 and Figure 3, support the interpretation that the history‐dependent internal state associated with coupled ferroelectric polarization relaxation and Cu‐ion‐related ionic dynamics provides a plausible physical mechanism for temporally correlated stochastic spiking.

**FIGURE 4:**
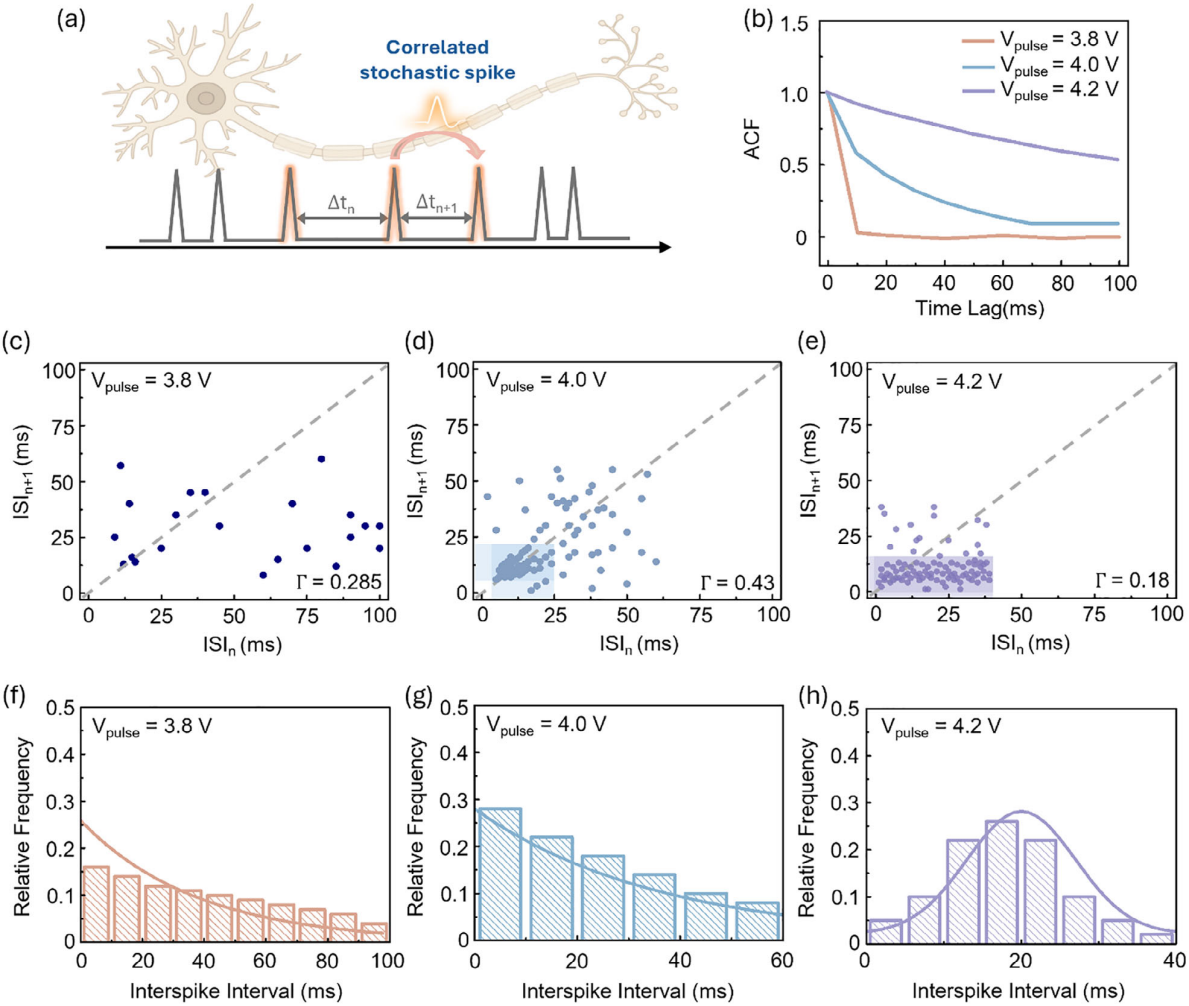
Temporal correlation analysis of stochastic spike. (a) Schematic illustration of temporal correlations between successive stochastic spike intervals. (b) ACF measured under V_pulse_ = 3.8, 4.0, and 4.2 V, respectively. (c–e) Correlation plots between ISI_n_ and ISI_n+1_ measured at V_pulse_ = 3.8, 4.0, and 4.2 V, respectively. The gray dashed line represents ISI_n_ = ISI_n+1_, serving as a reference for comparing successive spike intervals. Γ indicates the degree of temporal correlation between consecutive spike intervals. (f–h) ISI distributions measured at V_pulse_ = 3.8, 4.0, and 4.2 V, respectively.

### 2.5 Bayesian SSNN Based on Temporally Correlated Stochastic Spiking

To systematically evaluate how this neuron features voltage‐dependent spiking dynamics, particularly the temporally correlated stochastic firing behavior characterized in Figure 4, influence system‐level computational efficiency and computational performance, we constructed a hardware‐software co‐integrated Bayesian stochastic spiking neural network model. The experimentally extracted device characteristics, consisting of Γ and the baseline firing probabilities (*p_Base_*), were directly mapped onto the stochastic activation dynamics of the network (Table S1). Here, Γ indicates the degree of temporal correlation between successive spike events, whereas *p_Base_* reflects the intrinsic stochastic firing tendency of the neuron. *p_Base_* was derived by mathematically modeling the empirical switching dynamics as a time‐homogeneous Poisson process. By inverting the Poisson cumulative distribution function (CDF) from the time‐dependent cumulative spiking probability data (Figure S18), the intrinsic firing rate (λ), which is given by:

(3) $$\lambda =-\frac{\ln\left(1-P\left(t\right)\right)}{t}$$

The λ values were then averaged across the non‐saturated temporal window (0 <  *P*(*t*) < 1). The resulting *p_Base_* scaled exponentially with the applied voltage, rising from 0.0042 at 3.8 V to 0.0284 at 4.0 V, and finally saturating at 0.0669 at 4.2 V, reflecting the electric‐field‐driven enhanced of Cu^+^ ion migration dynamics. This voltage‐dependent characteristics indicates that stronger electric fields progressively lower the effective activation barrier for stochastic high‐conductance state formation, thereby facilitating spike generation. Therefore, *p_Base_* serves as a quantitative metric of the intrinsic electrical excitability of the neuron. A larger *p_Base_* indicates that the neuron becomes increasingly susceptible to stochastic spike generation even under identical input conditions, corresponding to a more electrically excitable and hyperactive neuronal state. Figure 5a illustrates the schematic of the SSNN processing MNIST handwritten digits, indicating the signal propagation behavior between our proposed neuron and conventional stochastic neurons. While conventional neurons generate temporally uncorrelated stochastic spikes without historical state dependence, our proposed neuron produces temporally correlated stochastic spikes. These temporally correlated spikes enable sequential temporal integration within the SSNN, enabling suppression of uncorrelated noise components during inference.

**FIGURE 5:**
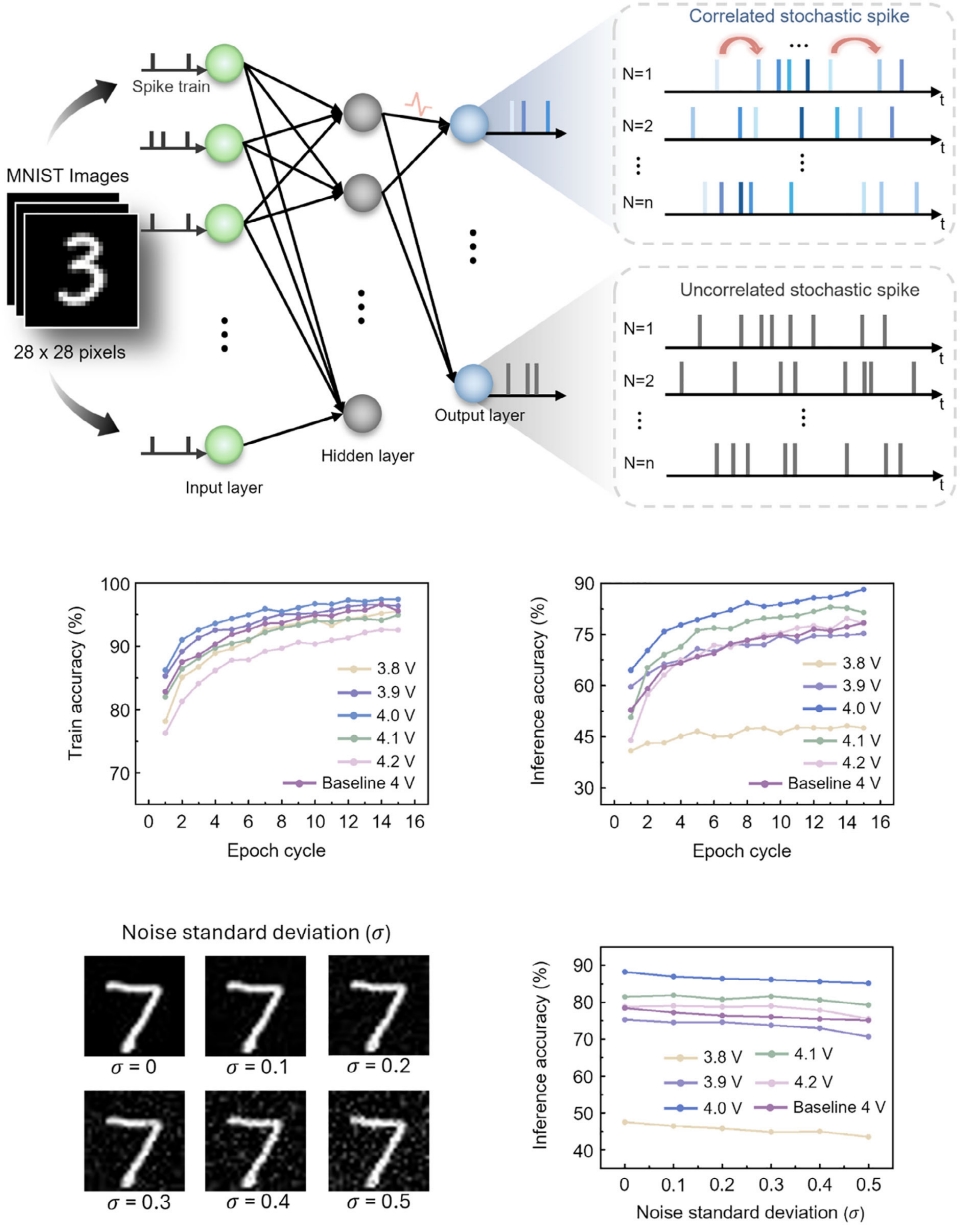
Bayesian SSNN simulation. (a) Schematic illustration of the Bayesian SSNN architecture employing correlated stochastic spikes for MNIST image classification. The right panels compare temporally correlated and uncorrelated stochastic spike behaviors. (b) Training accuracy measured under different voltage conditions. (c) Inference accuracy measured under different voltage conditions. (d) Noisy MNIST image examples under different noise standard deviation (σ) conditions. (e) Inference accuracy as a function of noise standard deviation. The correlated stochastic spike‐based SSNN maintains robust inference performance by suppressing temporally uncorrelated noise.

Using this network framework, we evaluated the baseline learning performance of over 15 training epochs under different operational voltages. As shown in Figure 5b, the training accuracy for all voltage conditions rapidly converges above 90% after only two epochs and eventually saturates near 95% at epoch 15. This result demonstrates that all voltage conditions provide sufficient representational capacity for fitting the training dataset under noise‐free learning conditions. To directly evaluate the contribution of temporal correlation, a memoryless stochastic‐neuron baseline was additionally evaluated under the same network architecture and training protocol. For a conservative comparison, the memoryless baseline was constructed based on the optimal 4.0 V operating condition while removing the history‐dependent temporal correlation. As shown in Figure 5b, the memoryless stochastic neuron exhibits a generally comparable learning convergence, indicating that the removal of temporal correlation does not fundamentally prevent network training. However, it is noted that significant differences emerge during the inference task, as shown in the inference accuracy as a function of epoch cycles in Figure 5c. The optimal voltage of 4.0 V achieves the highest classification accuracy, approaching ∼90%, whereas other operational voltages result in substantial degradation in inference performance. Importantly, the memoryless baseline shows lower inference accuracy than the temporally correlated CIPS neuron at 4.0 V, supporting the contribution of history‐dependent stochastic dynamics to inference performance. On the other hand, at the low voltage condition of 3.8 V, the inference accuracy remains limited to approximately 45%–50% throughout the training epoch. This degraded performance results from excessively sparse stochastic spike generation caused by the extremely low baseline firing probability (*p_Base_* = 0.0042). Due to infrequent transitions into the high‐conductance state, essential feedforward spike information fails to propagate reliably through the hidden layers. Consequently, the network behaves similarly to an excessively over‐regularized stochastic dropout system in which critical feature information is continuously lost during signal propagation. However, when the operational voltage of the neuron increases toward 4.2 V, the inference accuracy decreases significantly compared with the optimal 4.0 V condition, reaching approximately 75%. The excessively high firing probability (*p_Base_* = 0.0669), together with the diminished temporal correlation (Γ = ‐0.0122), drives the neuron into an electrically overexcited regime. In this condition, excessive stochastic spike activity continuously generates redundant background spikes that are weakly correlated with meaningful input features, thereby degrading the inference performance of the neural network.

To rigorously evaluate the fault tolerance and robustness of the trained SSNN under noisy environments, the input image tensors were subjected to pixel‐wise additive white Gaussian noise. Figure 5d exhibits representative examples of the MNIST handwritten digit “7” under systematically increased noise standard deviations (σ = 0.0, 0.1, 0.2, 0.3, 0.4, and 0.5). At the highest noise level (σ = 0.5), the structural features of the digit become substantially obscured by stochastic background perturbations. The inference accuracy of the trained SSNN as a function of σ is summarized (Figure 5e). The SSNN shows a distinct voltage‐dependent operational regime in terms of environmental noise robustness. The highest robustness to noise is achieved at the optimal pulse voltage of 4.0 V. Under this condition, the SSNN maintains stable inference accuracy above 87% even at the highest noise intensity (σ = 0.5). This robust behavior originates from the coexistence of the maximum positive ISI correlation (Γ = 0.4315) and the optimized baseline firing probability (*p_Base_* = 0.0284) at 4.0 V. At this intermediate voltage regime, the optimized firing probability establishes an effective activation threshold that is sufficiently sensitive to meaningful signal features while remaining robust against low‐level perturbations. Simultaneously, the strong temporal correlation enables sequential reinforcement of correlated structural features over the inference window while suppressing temporally uncorrelated environmental noise. The memoryless baseline at 4.0 V also exhibits lower inference accuracy under input noise than the temporally correlated CIPS neuron. This comparison indicates that temporal correlation provides additional robustness beyond stochastic firing alone.

In contrast, operation outside this optimized regime results in severe degradation in noise robustness. The 3.8 V condition exhibits persistently poor inference performance across all noise conditions because of insufficient spike generation and unreliable signal propagation. Meanwhile, at 4.2 V, the inference accuracy decreases rapidly with increasing σ. The excessively high firing probability at 4.2 V indicates that the neuron enters an electrically hypersensitive regime. As a result, even weak background perturbations are amplified into excessive stochastic spike activity, overwhelming the meaningful classification signals. These results demonstrate that the optimal operating condition results from a balance between stochastic excitability and temporal correlation dynamics. Taken together with the memoryless control, these results indicate that robust SSNN operation requires not only an appropriate stochastic firing probability but also the history‐dependent temporal dynamics provided by the CIPS neuron. Within this optimized voltage, the proposed neuron simultaneously achieves reliable signal propagation, temporal noise suppression, and energy‐efficient stochastic computation, establishing a robust neuromorphic hardware framework for probabilistic edge‐computing applications.

Table 1 compares previously reported hardware stochastic neurons in terms of hardware characteristics and network performance. From a hardware perspective, unlike previous stochastic neurons that primarily rely on random switching events, the proposed CIPS‐based neuron exhibits history‐dependent stochastic spiking and enables hardware synapse‐neuron integration with the FeFET synapse. From a network perspective, these hardware characteristics are incorporated into a Bayesian SNN, achieving an inference accuracy of 90.6% while maintaining over 87% accuracy under noise conditions (σ = 0.5).

**TABLE 1:** Comparison of hardware characteristics and network performance of stochastic neurons.

	Hardware characteristics			Network performance			
Device structure	Origin of stochasticity	History‐dependent spiking (O/X)	Hardware synapse‐neuron integration	Bayesian SNN simulation	Inference accuracy (%)	Inference accuracy under noise	Refs.
Pt/CuS/GeSe/Pt	Cu^2+^ ion migration and filament formation	X	X	O	97.0	X	[4]
Cu‐Ta/IGZO/TiN Schottky diodes	Cu‐ln/O nucleation	X	X	X	N/A	N/A	[12]
Pt/VO_2_/Pt	VO_2_ switching	X	X	X	N/A	N/A	[26]
Au/Pd/1T‐TaS_2_/SiO_2_	CDW domain reconfiguration	X	O	X	N/A	N/A	[27]
Three‐terminal perpendicular MTJ	Thermal fluctuation magnetization switching	X	X	X	92.4	X	[28]
Au/CIPS/Au	Cu^+^ ion hopping	O	O	O	90.6	> 87% (σ = 0.5)	**This work**

## 3 Conclusion

In this study, we demonstrate a SSNN by integrating an α‐ln_2_Se_3_/h‐BN/CIPS FeFET synapse with an Au/CIPS/Au neuron. Unlike conventional memoryless stochastic neurons, the proposed neuron generates stochastic spikes through the evolution of internal ferroelectric polarization states, giving rise to intrinsic temporal correlations and history‐dependent dynamics. Polarization‐state evolution and incomplete polarization relaxation are associated with the formation of a history‐dependent internal state, which continuously influences subsequent stochastic switching events and provides a plausible physical mechanism for temporally correlated stochastic spiking. Notably, the strongest temporal correlation was observed in the intermediate stochastic regime, with a maximum Pearson correlation coefficient of Γ = 0.43. Furthermore, we implemented an SSNN employing the proposed temporally correlated stochastic neuron and demonstrated that correlated stochastic dynamics can be effectively exploited for neural network computation. The resulting SSNN achieved an inference accuracy of approximately 90% and maintained over 87% accuracy under severe noise conditions (σ = 0.5). These results demonstrate that stochasticity, memory, and temporal correlation can coexist within a neuron and extend the functionality of conventional memoryless stochastic neurons. This work provides a pathway toward next‐generation probabilistic neuromorphic systems capable of intrinsic temporal information processing through temporally correlated stochastic spiking.

## 4 Methods Section

### 4.1 Device Fabrication

The CIPS flakes (HQ graphene) were mechanically exfoliated onto polydimethylsiloxane (PDMS) films and sequentially transferred onto a cleaned SiO_2_/Si substrate using the dry transfer method. The electrodes were patterned by electron beam lithography (EBL) over the heterostructure, and Au metal layers were deposited using an electron beam evaporator (schematically presented in Figure S1).

### 4.2 Device and Material Characteristic

An optical microscope (OM, Olympus, BX51M) was used to observe the size and fabricated devices. The thickness and topography of the device were measured using atomic force microscope (AFM, Park Systems, NX‐10) in noncontact mode. The PFM measurements were performed using an AFM system that was equipped with Cr/Pt coated conductive tips (Multi75E‐G, BudgetSensors, nominal spring constant k = ≈3 Nm^−1^). All electrical measurements were performed at room temperature using a probe station under ambient conditions with a Keithley 4200‐SCS system.

For evaluating the electrical pulse characteristics of the devices, a probe station, a semiconductor parameter analyzer (Keysight 4156B), and a pulse generator (Keysight 81110A) were utilized. All measurements were performed at room temperature. The cross‐sections were observed using TEM (JEOL, TEM2100F) at an accelerating voltage of 200 kV.

For the device‐to‐device statistical analysis, 50 functional devices were evaluated. The device yield was approximately 60%. Functional devices were defined as those exhibiting both ferroelectric switching and stochastic spiking behavior, and only devices satisfying these criteria were included in the device‐to‐device statistical analysis.

### 4.3 Extraction of the Pearson Correlation Coefficient for SSNN Simulation

To systematically bridge the physical characteristics of the 2D ferroelectric CIPS neuron to the neural network simulation, the temporal memory effect was quantitatively extracted from the experimental datasets. Utilizing the raw distribution points of consecutive spike intervals (ISI_n_and ISI_n + 1_), the Γ was calculated across the entire operational pulse voltage spectrum (3.8 to 4.2 V). The correlation analysis mathematically evaluated the linear dependence between successive stochastic spiking events using the following formulation (2). Where *n* represents the total number of consecutive spike pairs, and $\bar{\mathrm{IS}I_{n}}$ and $\bar{\mathrm{IS}I_{n+1}}$ denote the arithmetic means of the respective interval distributions. This calculated coefficient established a quantitative baseline representing the device's capacity to retain past internal polarization states and modulate subsequent spike generation based on its historical state.

### 4.4 Extraction of Baseline Firing Probability for SSNN Simulation

To establish the reference state of the neuron before incorporating temporal dependencies, the continuous intrinsic firing rate (λ) was uncorrelated prior‐free baseline activation was derived. Assuming a stochastic switching initiation under a zero‐activity background, the time‐series data of cumulative spiking probability (*P*(*t*)) was compiled for each pulse voltage condition. To isolate the pure baseline properties, the time‐homogeneous Poisson cumulative distribution function was used as the inversion proxy to extract λ computed as follows formulation (2). To ensure statistical stability and mitigate temporary experimental fluctuations, the calculated λ values within the non‐saturated probability boundaries (0< *P*(*t*) < 1) were averaged across the entire temporal evolution profile, establishing the definitive *p_Base_* parameter for each pulse voltage configuration. Crucially, while this baseline parameter itself assumes an initial memoryless state, the subsequent network framework superimposes the Γ to fully restore the temporal memory effects of the physical device.

### 4.5 Simulation of SSNN Noise Robustness

To evaluate system‐level fault‐tolerance, the hardware‐calibrated parameters (Γ and *p_Base_*) were integrated into the SSNN benchmarked against the MNIST dataset. The input pipeline processed gray‐scale MNIST images (28 × 28 pixels). External noise was modeled by adding pixel‐wise white Gaussian noise, with the noise standard deviation (σ) scaled from 0.0 to 0.5:

$$X_{noisy}=X_{normalized}+\eta \cdot \sigma$$

where $\eta \in R^{B\times 1\times 28\times 28}$ is a random tensor from a standard normal distribution, and *B* = 512 denotes the mini‐batch size. The network architecture was structured as a fully connected feedforward topology comprising three distinct functional layers: an input encoding layer (*N_in_* = 784 neurons), a single hidden processing layer (*N_hidden_* = 256 neurons), and an output classification layer (*N_output_* = 10 neurons). During inference task, the noise‐corrupted images were flattened into a 784‐dimensional vector and duplicated across 150 simulation time‐steps (*T* = 150), forming a temporal input tensor $S_{in}\in R^{T\times B\times 28\times 28}$. Within the temporal loop (*t*  =  1 to *T*), feedforward signals propagated through synaptic weight matrices linking the layers sequentially. The stochastic firing function of each neuron was directly governed by the hardware parameters. The *p_Base_* defined the stochastic switching threshold under zero prior activity. If a neuron fired a spike at time‐step *t* − 1, its firing probability at time‐step *t* was dynamically updated by adding the positive Γ to incorporate temporal memory feedback. This framework enabled temporal integration. Uncorrelated, high‐frequency spikes from the Gaussian noise were statistically suppressed by the historical state dependencies, whereas temporally correlated signal pathways representing true digit features were reinforced. Output spikes were accumulated over 150 time‐steps into a count vector $C\in R^{B\times 10}$, and final predictions were determined via a global argmax operation over *C* using the 10,000 testing samples.

## Author Contributions

**Hanseo Jeon**: conceptualization, investigation, writing – original draft. **Ui Do Ji**: conceptualization, investigation, writing – original draft. **Sangmin Lee**: investigation, methodology, validation. **Gihyeon Bhin**: investigation, methodology, validation. **Chang Yong Park**: investigation, methodology, validation. **Ji‐Sang Park**: conceptualization, investigation, writing – original draft. **Byungchul Jang**: conceptualization, investigation, writing – original draft. **Sungjoo Lee**: conceptualization, investigation, writing – original draft, supervision, funding acquisition.

## Conflicts of Interest

The authors declare no conflicts of interest.

## Supporting information




**Supporting File**: advs78300‐sup‐0001‐SuppMat.docx.

## Data Availability

The data that support the findings of this study are available from the corresponding author upon reasonable request.
