# Supplementary material for: Temporally Correlated Stochastic Spiking Neural Networks Enabled by 2D Ferroelectric Synapse‐Neuron Integration
Source: Adv Sci (Weinh). 2026 Oct 14:e78300. Online ahead of print. doi: 10.1002/advs.78300 (PMC13669219; doi:10.1002/advs.78300)
Supplement: Supplementary file 1 [file ADVS-9999-e78300-s001.docx]

**Supplementary Information**

**Temporally Correlated Stochastic Spiking Neural Networks Enabled by 2D Ferroelectric Synapse-Neuron Integration**

Hanseo Jeon^1,2†^, Ui Do Ji^3†^, Sangmin Lee^1,2^, Gihyeon Bhin^1,2^, Chang Yong Park^1,2^, Ji-Sang Park^1,2*^, Byungchul Jang^3*^ and Sungjoo Lee^1,2*^

^1^SKKU Advanced Institute of Nanotechnology (SAINT), Sungkyunkwan University, Suwon, Korea

^2^Department of Nano Science and Technology, Sungkyunkwan University, Suwon, Korea

^3^School of Electronic and Electrical Engineering, Kyungpook National University, Daegu, Korea

Table of contents

Supplementary Table S1. Experimentally extracted stochastic neuron parameters used for SSNN modelling

Supplementary Figure S1. Synapse-Neuron interfacing via load resistor

Supplementary Figure S2. Fabrication process of Neuron and Synapse

Supplementary Figure S3. CuInP_2_S_6_ Neuron and α-ln_2_Se_3_/h-BN/ CIPS FeFET Synapse Device

Supplementary Figure S4. Temperature- and frequency-dependent hysteresis loops of CIPS

Supplementary Figure S5. PFM switching spectroscopy of CIPS

Supplementary Figure S6. Multiple repeated measurements of the I-V characteristic of CIPS

Supplementary Figure S7. EDS mapping of CIPS flake

Supplementary Figure S8. Stochastic polarization revealed by repeated PFM measurements

Supplementary Figure S9. Neuron device current fluctuations under variable voltage conditions

Supplementary Figure S10. LTP/LTD characteristic of the FeFET synapse

Supplementary Figure S11. Retention and endurance characteristics α-ln_2_Se_3_/h-BN/ CIPS FeFET

Supplementary Figure S12. Device-to-device voltage dependent stochastic spike probability Supplementary Figure S13. Temperature dependent on stochastic spike probability

Supplementary Figure S14. Voltage-dependent first-spike latency of the CIPS stochastic neuron

Supplementary Figure S15. Voltage-dependent switched polarization retention measurement

Supplementary Figure S16. Device-to-device ISI

Supplementary Figure S17. Pulse-width dependent temporal correlation of the CIPS neuron

**Supplementary Figure S18. Time dependent cumulative spike probability under different applied voltages**

**Supplementary Table S1. Experimentally extracted stochastic neuron parameters used for SSNN modelling**

| **Pulse (voltage)** | $\boldsymbol{\Gamma}$ | $\boldsymbol{p}_{\boldsymbol{Base}}$ |
| --- | --- | --- |
| 3.8 | -0.034437105 | 0.004195425 |
| 3.9 | 0.343384272 | 0.010633601 |
| 4.0 | 0.431534271 | 0.028437733 |
| 4.1 | 0.242780237 | 0.048944861 |
| 4.2 | -0.012229215 | 0.066903885 |

**Supplementary Figure S1. Synapse-Neuron interfacing via load resistor**

Figure S1 shows the interfacing circuit between FeFET synapses and CuInP_2_S_6_ neuron. The current output of the FeFET synapse is converted into a voltage (V_N_) through a load resistor (R_load_), which serves as the input to the CIPS neuron. The introduction of the V_N_ node enables controlled signal transduction while accounting for potential input/output and impedance mismatch between the synapse and neuron devices.

**Supplementary Figure S2. Fabrication process of Neuron and Synapse**

Figure S2 shows the device fabrication process. To fabricate the bottom electrode, polymethyl methacrylate (PMMA) was spin-coated onto a p^++^ Si/SiO_2_ substrate at 450 K and 2000 rpm for 5 s, followed by 950 K and 4000 rpm for 35 s. The bottom electrodes were then experimented by electron beam lithography, and Au (20 nm) was deposited using an electron beam evaporator in a high-vacuum chamber (5 $\times$ 10^-7^ Torr). Subsequently, a CIPS flake was transferred onto the substrate via a dry transfer process inside a glove box. Finally, the top gate electrode was fabricated by depositing Au (80 nm) using the same deposition process.

Supplementary Figure S3. CuInP_2_S_6_ Neuron and α-ln_2_Se_3_/h-BN/CIPS FeFET Synapse device

Figure S3 shows (a) optical microscopy (OM) image of CIPS device, Yellow dashed lines are the Au electrode region, and the blue line is the CIPS flake region. (b) optical microscopy image of α-ln_2_Se_3_/h-BN/CIPS FeFET. Yellow dashed lines are the Au electrode region, red dashed line is α-ln_2_Se_3_, gray dashed line is h-BN and the blue line is the CIPS flake region. (c) height profile of a CIPS device and α-ln_2_Se_3_/h-BN/CIPS FeFET. Thickness profile by atomic force microscope (AFM) along the white line.

**Supplementary Figure S4. Temperature- and frequency-dependent hysteresis loops of CIPS**

Figure S4 presents the hysteresis loop of the Au/CIPS/Au device, obtained from hysteresis loops measurement. The polarization was calculated by integrating the measured current during a voltage sweep from -10 V to +10 V. (a) hysteresis loops measured at temperatures ranging from 220 to 310 K. (b) hysteresis loops measured at frequencies ranging from 50 Hz to 2 kHz. Hysteresis with a coercive voltage of 1.8 V indicates ferroelectric switching. The existence of remnant polarization at zero bias confirms the non-volatile ferroelectric nature of CIPS, which provides the physical basis for state-dependent switching and enables stochastic neuron operation.

**Supplementary Figure S5. PFM switching spectroscopy of CIPS**

Figure S5 shows the PFM switching characteristics of CIPS. (a) PFM phase hysteresis loop and (b) PFM amplitude hysteresis loop as a function of DC bias.

Supplementary Figure S6. Multiple repeated measurements of the I-V characteristic of CIPS

Figure S6 shows the I-V characteristic of CIPS devices. Stable rectifications were observed even after repeating 75 cycles. I-V characteristic of CIPS devices measured by sweeping the bipolar voltage from -5 V to +5 V.

**Supplementary Figure S7. EDS (Energy-Dispersive X-ray Spectroscopy) mapping of CIPS flake**

Figure S7 shows OM images and EDS elemental mapping of exfoliated CIPS flakes. The dashed red line in the OM images denotes the region analyzed by EDS. The spatial distributions of Cu, ln, P, and S are uniform across the entire flake, indicating homogeneous stoichiometry without noticeable compositional variation. Such uniform elemental distribution ensures reliable ferroelectric behavior and consistent device performance.

**Supplementary Figure S8. Stochastic polarization revealed by repeated PFM measurements**

Figure S8 presents repeated piezo-response force microscopy (PFM) phase measure of CIPS flake, along with corresponding phase distribution histograms. Under identical conditions (4 V), the polarization states exhibit stochastic variations while preserving similar domain ratios, reflecting an intrinsic stochastic switching process. Such behavior arises from Cu^+^ ion dynamics and from the basis for state dependent polarization evolution in the device.

**Supplementary Figure S9. Neuron device current fluctuations under variable voltage conditions**

Figure S9 shows the current fluctuation under various voltage conditions and detailed spike probability of the sigmoid curve at CIPS device. The current value was obtained by repeating the measurement 500 cycles with varying program voltages.

Supplementary Figure S10. LTP (long-term potentiation)/LTD (long-term depression) characteristics of the FeFET synapse

Figure S10 shows the LTP and LTD characteristics of the α-ln_2_Se_3_/h-BN/CIPS FeFET synapse measured under consecutive potentiation and depression pulses. The gradual conductance modulation demonstrates the feasibility of the FeFET device as a synaptic weight for hardware SSNN implementation.

**Supplementary Figure S11. Retention and endurance characteristics α-ln_2_Se_3_/h-BN/CIPS FeFET**

Figure S11 presents the retention and endurance characteristics of 4 devices measured under programming/erasing pulses at ±10 V, ±5 V and ±4 V. The retention behavior was observed through the temporal variation of I_DS_, while the endurance performance was evaluated over repeated programming/erasing cycles.

**Supplementary Figure S12. Device-to-device voltage dependent stochastic spike probability**

Figure S12 shows the voltage dependent spike probability measured from CIPS stochastic neuron 50 devices. (a) For devices with an 80 nm thick CIPS layer, the voltage spike probability characteristic is shown, representative device to device variations at each voltage point along with a representative sigmoid curve. (b) The distribution of the sigmoid slope (k) extracted from the same 50 devices is presented.

**Supplementary Figure S13.** **Temperature dependent on stochastic spike probability**

Figure S13 shows the temperature dependence of stochastic spike probability. As the temperature increases, the probability of spikes gradually increases. Error bars represent the standard deviation extracted from repeated 10 cycles measurements at each bias.

**Supplementary Figure S14. Voltage-dependent first-spike latency of the CIPS stochastic neuron**

Figure S14 shows the first-spike latency of the CIPS stochastic neuron according to the applied pulse voltage. The first-spike latency decreases from 87 ms at 3.8 V to 30 ms at 4.0 V and 7 ms at 4.2 V, indicating a faster firing response with increasing pulse voltage.

**Supplementary Figures S15. Voltage dependent switched polarization retention measurement**

Figure S15 shows the retention characteristics of switched polarization measured after applying 50 pulses at 3.8 V, 4.0 V, and 4.2 V to an Au/CIPS/Au device with an 80 nm thick CIPS layer. The initial switched polarization is approximately 3.5~3.6 $\mu C/\mathrm{cm}^{2}$ for all voltages. At 4.2 V, the switched polarization remains nearly constant up to 1000 s. At 3.8 V and 4.0 V, the initially formed polarization state is maintained for a certain period, followed by a gradual decrease.

**Supplementary Figure S16. Device-to-device ISI**

Figure S16 shows the correlation between consecutive inter-spike interval (ISI) across multiple devices. This consistent trend across devices confirms the reproducibility of correlated stochastic spiking behavior.

**Supplementary Figure S17. Pulse-width dependent temporal correlation of the CIPS neuron**

Figure S17 shows the ISI_n_, ISI_n+1_ distribution measured at 4 V under different pulse widths (5 ms, 50 ms, 100 ms). The Pearson correlation coefficient ($\Gamma$) decreases with increasing pulse width ($\Gamma$ ≈ 0.58 at 5 ms, $\Gamma$ ≈ 0.31 at 50 ms, and $\Gamma$ ≈ 0.19 at 100 ms).

**Supplementary Figure S18. Time dependent cumulative spike probability under different applied voltages**

Figure S18 presents the cumulative spike probability as a function of time under different applied voltages from 3.8 to 4.2 V. The cumulative spike probability exhibits voltage-dependent temporal evolution behavior.
